# Clustered microRNAs' coordination in regulating protein-protein interaction network

**DOI:** 10.1186/1752-0509-3-65

**Published:** 2009-06-26

**Authors:** Xiongying Yuan, Changning Liu, Pengcheng Yang, Shunmin He, Qi Liao, Shuli Kang, Yi Zhao

**Affiliations:** 1 Bioinformatics Group, Center for Advanced Computing Research, Institute of Computing Technology, Chinese Academy of Sciences, Beijing, PR China; 2 Graduate School of Chinese Academy of Sciences, Beijing, PR China; 3 Department of Parasitology, Zhongshan School of Medicine, Sun Yat-sen University, Guangzhou, PR China

## Abstract

**Background:**

MicroRNAs (miRNAs), a growing class of small RNAs with crucial regulatory roles at the post-transcriptional level, are usually found to be clustered on chromosomes. However, with the exception of a few individual cases, so far little is known about the functional consequence of this conserved clustering of miRNA loci. In animal genomes such clusters often contain non-homologous miRNA genes. One hypothesis to explain this heterogeneity suggests that clustered miRNAs are functionally related by virtue of co-targeting downstream pathways.

**Results:**

Integrating of miRNA cluster information with protein protein interaction (PPI) network data, our research supports the hypothesis of the functional coordination of clustered miRNAs and links it to the topological features of miRNAs' targets in PPI network. Specifically, our results demonstrate that clustered miRNAs jointly regulate proteins in close proximity of the PPI network. The possibility that two proteins yield to this coordinated regulation is negatively correlated with their distance in PPI network. Guided by the knowledge of this preference, we found several network communities enriched with target genes of miRNA clusters. In addition, our results demonstrate that the variance of this propensity can also partly be explained by protein's connectivity and miRNA's conservation.

**Conclusion:**

In summary, this work supports the hypothesis of intra-cluster coordination and investigates the extent of this coordination.

## Background

MicroRNAs (miRNAs) are small (22 nt) single-stranded non-coding RNA (ncRNA) molecules. They are processed from hairpin precursors of approximately 70 nt (pre-miRNAs), which in turn are extracted from primary transcripts (pri-miRNA) [1,2]. miRNAs can repress gene expression post-transcriptionally by binding to the 3' untranslated regions (3' UTRs) of their target mRNAs [3-5]. In animal genomes, miRNAs are found in various genomic locations; while most are located in intergenic regions, some are found to be hosted within the introns of pre-mRNAs or within longer ncRNA genes [6,7]. Interestingly, known miRNA genes, both hosted and non-hosted, are often observed to be clustered [8]. A cluster usually includes two or three miRNA genes, but larger clusters have also been identified, including a human *mir-17 *cluster comprising 6 miRNA genes and a human *mir-302 *cluster comprising 8 miRNA genes. Baskerville and Bartel found that miRNAs within 50 Kb are highly correlated in expression across 24 different human organs, indicating that 50 Kb might be used as preliminary definition for miRNA cluster [9].

Although miRNAs in a given cluster are usually phylogenetically related in sequence, many human clusters containing miRNAs without apparent sequence homology are also found [10]. A plausible but yet-to-be validated possibility is that the clustered miRNAs are functionally related by virtue of targeting the same gene or different genes in the same pathway [8,10-12]. Studies of individual cases have to some extent supported this hypothesis. For instance, *mir-15a-16 *cluster act as tumor suppressor genes in prostate cancer by controlling cell survival, proliferation and invasion [13]. In gastric cancer, *mir-106b *cluster and *mir-222 *cluster are upregulated and modulate cell cycle by targeting the Cip/Kip family proteins [14]. The frequently studied *mir-17-92 *cluster acts not only in tumor formation but also in development of heart, lungs, and immune system [15-18]. Three individual miRNAs of the *mir-379-410 *cluster are required for activity-dependent development of hippocampal neurons [19]. *mir-48 *and *mir-241 *of a *let-7 *family cluster function together to regulate developmental timing in *Caenorhabditis elegans *[20]. However, apart from these individual cases, the hypothesis has not yet been systematically investigated.

Here, we conduct a comprehensive analysis of the conservation and functional consequence of miRNA clustering. We show that clustered miRNAs have functional relatedness through co-targeting proteins in close proximity of PPI network.

## Results

### MiRNA clusters' construction and property

It has been reported that clusters may contain unrelated miRNAs [21,22]. But the frequency of such heterogeneity has not been comprehensively evaluated. Prior research [10] has identified the clustering of 326 registered human miRNA genes, using the strategy proposed by Altuvia et al. [23]. According to miRBase [24], the number of registered miRNA genes has doubled. Still more homologs of the known miRNAs are expected in human genome, especially when more and more miRNA genes derived from TEs (transcriptional elements) have recently been identified [25-27].

In order to analyze the genomic clustering of miRNAs as systematically and exhaustively as possible, we conducted a cross-species Blast search of known pre-miRNA sequences in the entire human, mouse and rat genomes. In rat genome we found 152 novel miRNA loci that are homologues of previously annotated human or mouse miRNAs, whereas the corresponding numbers of loci in the mouse and human genomes are 33 and 19, respectively (see Additional File 1). We clustered miRNA genes within 50 kb of each other according to Bartel et al. [9]. After filtering with RNA polymerase II promoter analysis software and expression profiles (for details, see Material and Methods), a total of 104 miRNA clusters representing 67 unique miRNA combinations have been identified in human genome. Clusters composed of only *hsa-mir-566 *were not included, since *hsa-mir-566 *is thought to be derived from repeat sequence *Alu*, which is pervasive in the human genome [28]. Similarly, 91 clusters representing 64 unique miRNA combinations were found in mouse (*mmu-mir-680 *excluded), and 56 miRNA clusters, all unique, were identified in rat.

In recent releases of miRBase, similar miRNA precursors are assembled into miRNA families (miFams) based on computational analysis and manual inspection. miRNAs in a family have been shown to be phylogenetically related [29,30]. We further mapped the miRNAs in our data to their families according to miRBase's assignment. As results, 55 distinct miRNA clusters were identified at family level in human genome, along with 51 in mouse, and 42 in rat (see Additional File 2). After mapping to families, 31 clusters have been found to be conserved among the three species, which take up 56% in human, 61% in mouse and 74% in rat (see Additional File 3).

Approximately half of the identified clusters are composed of multiple miRNA families and are denoted as miRNA hetero-cluster throughout this paper. On average, a hetero-cluster contains three miRNA families, and around half of the miRNA genes in a hetero-cluster do not belong to the main family (Table 1). Additionally, even miRNAs in the same family show sequence divergence and possibly target different proteins. This cluster heterogeneity is unlikely to merely derive from random mutation and retention. The fact that these clusters have been fixed in several modern animal genomes implies some evolutionary advantages of such a miRNA gene organization, which may provide an efficient internal mechanism for them to function in coordination [8,12,31].

**Table 1:** Heterogeneity analysis of the miRNA clusters.

	human	mouse	rat
HEC/TC	53/104	47/91	26/56

AFH	2.5	3.1	3.3

PNM	0.49	0.52	0.53

HEC – The number of hetero-clusters. TC – Total number of clusters. AFH – Average number of families in a hetero-cluster. PNM – Average percentage of miRNAs do not belong to the main family in each hetero-cluster.

### Functional Coordination on PPI network

Coordination among regulators from the transcriptional and miRNA regulatory layers has recently drawn great interests [32,33]. To reveal why non-homologous miRNAs are found in the same cluster, we analyzed their potential coordination in the context of PPI networks. Generally, the coordination of miRNAs in a cluster should be analyzed directly through justifying the relative proximity between their targets in the network. However, because of the incompleteness of credible PPI networks (e.g. Human Protein Reference Database, HPRD) and the low reliability of other integrated databases, we primarily chose to reverse the procedure to analyze whether proteins in close proximity incline to be regulated by miRNAs from the same cluster (henceforth referred to as "sc-miRNAs").

We first investigated directly interacting protein pairs. It has recently been reported that interacting protein pairs have a certain propensity to be regulated by the same miRNA [34]. There may exist a similar tendency that two interacting proteins are also regulated by sc-miRNAs. Sc-miRNAs can fulfil the same function through a complementary approach – by assembling two non-related miRNA loci in the same transcriptional unit. Before analysis, we prepare the data as follows:

We used HPRD as the golden standard positive (GSP, interacting protein pairs) dataset, and the dataset generated by Rhodes et al. [35] according to protein subcellular localization as the golden standard negative (GSN, non-interacting protein pairs) dataset. Comparisons of various miRNA target prediction programs suggest that TargetScan could achieve both high sensitivity and specificity [36,37]. We therefore employed TargetScan predictions for clustered miRNAs. For targets of a miRNA family, common targets shared by all its miRNA members were used. The number of miRNA sites is 9.7 ± 8.9 per transcript for GSP, and 10.1 ± 9.4 for GSN. To specifically evaluate the coordination among different miRNA families in a cluster, we constructed a family-represented version of hetero-clusters by mapping miRNAs in a cluster to their families, and then having redundancies removed (We will later denote these clusters as family-represented hetero-clusters and clusters before collapsing as original clusters). As a result, the 53 hetero-clusters have been collapsed to 37 family-represented hetero-clusters. To curb possible bias in the target prediction and isolate the effects of clustering, a control was generated by randomly reshuffling miRNAs (or families, when studying family-represented hetero-clusters) among the clusters.

**Figure 1:**
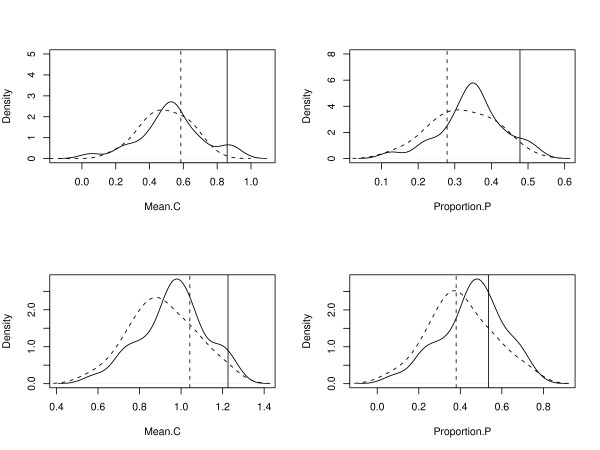
**The prevalence of coordinated regulation from sc-miRNAs**. The left column shows the average number of clusters that co-regulate a protein pair (Mean.C) and the right column shows the proportion of protein pairs under co-regulation (Proportion.P). The first row shows Mean.C and Proportion.P calculated on the four combinations of GSP or GSN with family-represented hetero-clusters (HC) or its 1000 randomizations (RC, Random cluster set). GSP-HC is shown in solid line, GSN-HC in dashed line, GSP-RC in solid curve and GSN-RC in dashed curve. The second row shows Mean.C and Percent.P calculated on the four combinations of GSP or GSN with original clusters or its 1000 randomizations.

Two measures were employed to assess sc-miRNAs' coordination: one measure is the number of clusters that regulate the two proteins, which reflects the strength of coordination; the other is the percentage of protein pairs that are regulated by sc-miRNAs, which reflects the range of coordination. We calculated the prevalence of miRNAs' coordination on the GSP and GSN datasets for real clusters and 1,000 random cluster sets. Interacting proteins are found to have a significantly higher tendency to be co-regulated by miRNAs from real than from randomly organized clusters (Figure 1). This tendency towards coordinated regulation was not observed for the GSN dataset, which justifies our study of sc-miRNAs' coordination in the context of PPI network. The large coordination measurements detected in random cluster set might be due to: 1) considerable noise exists in current protein-protein interaction data and miRNA target prediction; 2) when reshuffling 95 miRNAs families (contains redundancies, when a family appears in more than one cluster) in 37 hetero-clusters, there is a chance that two families in a real cluster are assigned to a random cluster again; meanwhile, miRNAs from the same family, which usually locate in one or two clusters, were dispersed into more clusters in randomization, and hence substantially increased the number of clusters they coincided.

Since the interacting pairs demonstrate stronger propensity to be regulated by sc-miRNAs than non-interacting pairs, we next asked whether there are some correlation between the propensity and the distance between two proteins in the network. The shortest paths were calculated between all the proteins in GSP dataset. We found a significant negative correlation between the number of clusters regulating the two proteins and the length of the shortest path between them in the PPI network (Spearman's rank correlation, correlation coefficient Rs = -0.24, P < 2.2e-16). This result suggests that distance in the PPI network is an important factor with respect to the coordinated regulation from sc-miRNAs, and lend further support to our previous finding.

We have also noticed the great variance in the number of clusters regulating an interacting protein pair. Still 50% of the directly interacting protein pairs are not co-regulated by miRNAs from same cluster, and the numbers of clusters that do regulate them vary from 1 to 14. Despite the noises and data incompleteness, directly interacting pairs are still likely to enjoy different levels of tendency. Therefore, we studied another factor, *connectivity*, that might influence this propensity.

Connectivity, the number of neighbours, is one of the most important properties of a protein node in a PPI network. In general, it quantitatively measures functional complexity and importance of a protein node in the PPI network. We counted all proteins' connectivity in HPRD network. The connectivity of proteins in GSP is 15.7 ± 19.3, and in GSN 11.3 ± 17.6. We added up the connectivity of the two proteins in each pair and discovered a positive correlation between connectivity sum and the number of miRNA clusters targeting them in GSP (Spearman's rank correlation, correlation coefficient Rs = 0.11, P = 3.136e-8, as detailed in Table 2). More interestingly, this positive correlation is two times stronger in GSP than GSN (Rs is only 0.06 in GSN-HC, which is close to a random control). Considering the major difference between GSP and GSN is that the shortest path is longer than 3 between GSN proteins in HPRD network, this result indicates that the sc-miRNAs preferentially act on protein pairs within close proximity, rather than those with greater connectivity (importance) in PPI network. It may explain why coordination has mainly been reported in regulating pathways.

**Table 2:** A positive correlation between the sum of each two proteins' connectivity and the number of clusters regulating them.

Spearman		GSP-HC	GSN-HC	GSP-RC	GSN-RC
Hetero-Clusters	Rs	0.11	0.062	0.055	0.031
	
	P	3.136e-8	0.002	0.006	0.217

Ori-Clusters	Rs	0.118	0.058	0.035	0.045
	
	P	2.952e-9	0.019	0.165	0.025

Rs and P-value were from Spearman's rank test. For the test, 2500 pairs were randomly sampled from GSP and GSN respectively. The last two columns show the results calculated on a random cluster set.

To see whether these observations are robust, the same analysis was performed with PicTar prediction set, the overlap of TargetScan and PicTar prediction set as recommended by Sethupathy [36], as well as miRanda prediction set with alignment score greater than 155 (see Additional File 4). They all gave very consistent results.

### Modularity of the targets

**Figure 2:**
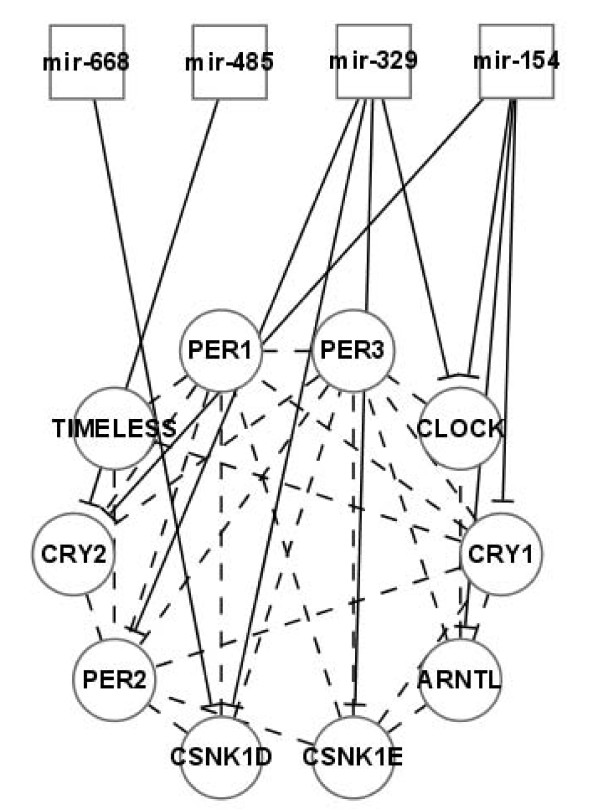
**A network community of ten proteins is co-regulated by four miRNA families in *mir-379 *cluster**. This network community is involved in *circadian rhythm *[KEGG pathway:*hsa04710*] and is co-regulated by nine miRNAs (*mir-494, mir-543, mir-495, mir-381, mir-539, mir-668, mir-485, mir-496*, and *mir-409*) from four families in *mir-379 *cluster. We mapped these miRNAs to their families for the simplicity of illustration.

A module in networks is a local structure characterized by more internal than external links. Since our findings suggest that sc-miRNAs favour coordinated regulation of local regions in PPI network, does sc-miRNAs' target gene set demonstrate some modularity? Xu and Wong [38] have found three miRNA clusters involved in regulating 15 signaling pathways by filtering mouse Biocarta pathway data. Pathway has been recognized as a common form of modularity in network analysis. Here, we explored another form of modularity, *network community*, in sc-miRNAs' targets with respect to HPRD network. Community structure in networks means the appearance of densely connected groups of vertices, with only sparser connections between groups [39]. We have found 32 network communities of size no less than 10 from HPRD network by joining cliques with size 3 to 7 together, using CFinder 2.0 [40]. We selected those network communities with at least 50% proteins targeted by a miRNA cluster and at most 40% regulated by any member of the cluster to guarantee that they are co-regulated by the cluster rather than any single member. For the 8 community-cluster pairs we obtained, the P-value was calculated based on the overlap of each community with the targets of one thousand simulated miRNA clusters of the same size. Finally, we obtained 5 community-cluster pairs with P < 0.03 (FDR < 0.2, Table 3). Among them, *mir-17 *cluster's role in transcription regulation through this protein community is well-known [41,42]. Xie et al. recently reported *mir-512 *cluster's involvement in histone acetylation in embryonic stem cells [43]. Another interesting one is *mir-379 *cluster. It targets a network community of ten proteins that are involved in circadian rhythm (Figure 2). Although there is no experimental report on it, circadian rhythm is modulated by neural system while *mir-379 *cluster is brain-specific and is required for dendritogenesis [19].

**Table 3:** Significant network communities that are enriched with target genes of miRNA clusters.

Chr	Cluster	Network Community	Function
3(-)	*let-7*	*ACTA1*, *DRP2*, *SNTA1*, *DMD*, *DAG1*, *SNTB2*, *UTRN*, *SNTG1*, *SNTG2*, *KCNJ12*, *SNTB1*, *PGM5P1*, *DTNA*, *DTNB*, *DGKZ*	muscle development and contraction

13(+)	*mir-17*	*AR, ESR1, RB1, TP53, CREBBP, EP300, JUN, STAT3, NCOA1, SMAD4, SMAD2, SMAD3, BRCA1, STAT1, TRIP4, RELA, CCND1, SP1*	positive regulation of transcription

14(+)	*mir-379*	*ARNTL, PER1, TIMELESS, CRY1, CLOCK, PER3, CRY2, PER2, CSNK1D, CSNK1E*	circadian rhythm

19(+)	*mir-512*	*HDAC2, RBBP7, RBP1, SAP30, HDAC1, ING1, RBBP4, BRMS1, SIN3A, BRMS1L, C2ORF59*	histone deacetylase

X(-)	*mir-450*	*TP53, CUL5, COPS3, GPS1, COPS2, COPS8, COPS6, COPS5, COPS4, COPS7A*	cancer suppressor *p53 *with *COP9 *signalosome

Although our work mainly focus on protein-protein interaction data, for it is more complete and more suitable for global analysis, we re-ran our analysis with KEGG human pathway data, and presented the results in Additional File 4.

### Coordination and conservation

Cluster provides a neat mechanism to transcribe many cooperative miRNAs simultaneously. It could hardly be imaged that this exquisite coordination has been generated in a single event; rather, it should have been consolidated through a long period of evolutionary process. To investigate the relationship between coordination and conservation, we have to calculate the target interactions between miRNAs in a cluster.

**Figure 3:**
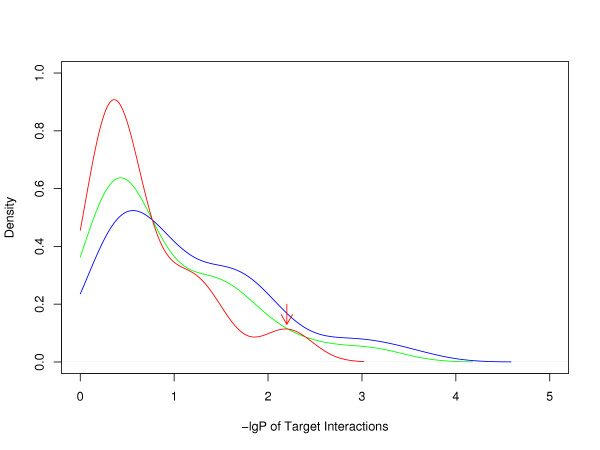
**Coordination among non-conserved sc-miRNAs (red curve) is weaker than that among conserved sc-miRNAs (blue curve)**. -lgP of Target interactions between conserved and non-conserved sc-miRNAs is shown in green curve. The arrow-pointed peak in non-conserved miRNAs was formed due to coordination within rapidly evolving non-conserved families in *mir-379 *cluster.

A comparison between human, mouse and rat miRNA clusters enables us to classify miRNAs in hetero-clusters into two categories: one is conserved miRNAs, which are observed in human, mouse and rat clusters; the other is non-conserved miRNAs, which exist in human clusters but are unobtainable in mouse or rat clusters. We counted the target interactions irredundantly within and between non-conserved miRNAs and conserved miRNAs. miRNA pairs with targets overlapping more than 100 within HPRD-recorded proteins were excluded because their mature sequences are so similar as to be suspected of having identical function. We calculated the empirical P-value of each miRNA pair's number of target interactions by 10,000 times randomization of miRNA-target protein association. The negative logarithm of P-value (-*lgP*) was then used to assess the coordination. The greater -*lgP *is, the stronger the coordination is. As results, 33.3% of conserved miRNAs pairs have been found to cooperate significantly in regulating PPI network (-*lgP *> 1.5, FDR < 0.1), which comports with our previous finding of intra-cluster coordination. Coordination within non-conserved miRNAs, however, is much weaker than that within conserved ones in a cluster (Figure 3, one-sided two-sample Kolmogorov-Smirnov test: D = 0.34, P = 1.38e-11). In other words, conserved miRNAs are generally more cooperative with each other than non-conserved ones in a cluster. The result is consistent on miRanda target set.

Although results in previous section indicate that miRNAs in a cluster may co-regulate proteins in close proximity of PPI network, they provide no information on each miRNA cluster's coordination. Here, those highly coordinated miRNA pairs (-*lgP *> 1.5), conserved and non-conserved, come from 17 (30.1%) hetero-clusters. We have found literature support for the function of 15 of them, all listed in Additional File 2. Thus it can be estimated that at least 30% of the miRNA hetero-clusters we can find evidence in current HPRD network for their intra-cluster coordination.

## Discussion

Recently, global analysis of miRNA targets in the context of sets of genes or PPI network has drawn great interests [34,38,44-47]. It could also provide important insights into how miRNAs in a cluster cooperate with each other. In this work, we adopted a protein-centered perspective to start our analysis. We fixed a protein set and analyzed the relationship of miRNAs that target them. This perspective has advantages in avoiding the incompleteness of protein-protein interaction data, and allowing us to analyze miRNAs' functionality according to the features of their targets.

By using the protein-centered perspective, we observed that directly interacting proteins incline to be regulated by miRNAs in the same cluster. The closer these proteins are in the PPI network, the more likely the targeting miRNAs are located in the same cluster. Proteins usually fulfill certain functions by means of interaction. If their functions are temporarily not needed, then their expressions should be reduced simultaneously. miRNAs in a cluster transcribed as polycistron would provide a competent and efficient mechanism to achieve this goal. Our further analysis reveals that connectivity is another factor that matters only when proteins interact or are at least close in PPI network. Interacting proteins, both with great connectivity, usually connect two functional protein groups. They would be under stronger selective pressure to consent to a miRNA cluster's regulation. If one of them was not regulated in time, the module it involved would likely be still in function, and the coordination of the whole system would be greatly impaired.

The finding that distance is more important than connectivity suggests that sc-miRNAs preferentially co-regulate proteins in close proximity of a PPI network. However, this may also be due to the fact that PPI network are mainly gathered from high-throughput experiments which focus on physical interactions, such as yeast two hybrid system and tandem affinity purification. Therefore, for two proteins with long distance in PPI network, even their concurrence in time and space could not be guaranteed. To overcome this limitation, other information, such as signaling network or transcriptional regulatory network, would be needed [46,47].

## Conclusion

This study supports the putative hypothesis of internal coordination among sc-miRNAs to regulate downstream biological networks. The linkage of sc-miRNAs' functional coordination and their targets' topological features we found highlights the potential to further investigate their subtle relationship.

## Methods

### Data sources

#### Datasets of PPI

Gold standard positive dataset: all the information in the Human Protein Reference Database (HPRD) has been manually extracted from the literature by expert biologists who read, interpret and analyze the published data. It contains 38,167 distinct interactions among 9,465 proteins [48]. Our analysis focused exclusively on the giant connected component of 9,134 proteins and 31,909 interactions. The remaining proteins were tiny clusters with sizes between two and eight. According to TargetScan's prediction, 8,264 of the 9,134 proteins are targeted by miRNAs. 6,737 of them are targeted by clustered miRNAs, and 4,835 are targeted by miRNAs that appear in hetero-clusters.

Gold standard negative dataset: it was generated by Rhodes et al. [35], which includes all possible pair-wise combinations between two sets of proteins that are assigned a subcellular localization of the plasma membrane (1,397 proteins) and the nucleus (2,224 proteins), respectively, by the Gene Ontology (GO) Consortium. There are 2633 overlapping proteins between GSP and GSN.

#### miRNA target prediction

TargetScan's release 4.2 was downloaded from TargetScan site . miRanda's prediction for 418 human miRNAs in microRNA registry release 10.0 were downloaded from microRNA.org Jan 2008 release with align score higher than 155. PicTar prediction was downloaded from UCSC genome browser PicTar miRNA track.

### Computational framework

#### Determination of miRNA clusters

We download all pre-miRNA sequences of *Homo sapiens *(hsa, 533 miRNAs), *Mus musculus *(mmu, 442 miRNAs) and *Rattus norvegicus *(rno, 285 miRNAs) from the miRNA registry release 10.0 , and blast them against the whole genome of human, mouse and rat. Only the hits with 90% coverage and 90% identity were selected. All miRNA genes whose Blast distance is smaller than 100 bp were treated as one locus. According to Baskerville and Bartel [9], we considered loci within 50 kb of each other as belonging to the same cluster. miRNAs in a cluster were further screened using following filters:*RNA polymerase II filter*: Clusters were classified as intronic or intergenic according to their hostages. miRNA genes located at the introns or UTRs of protein-coding genes are transcribed together with their host genes, whereas miRNA genes dispersed in an intergenic region are generally believed to be transcribed by RNA polymerase II [49]. Therefore, we screened the regions surrounding intergenic miRNA clusters using the following RNA polymerase II promoter analysis software: Promoter Scan  for predicting promoter regions based on scoring homologies with putative eukaryotic Pol II promoter sequences, Promoter Prediction 2.0  for predicting transcription start sites of vertebrate Pol II promoters in DNA sequences, and NNPP (neural network promoter prediction)  for predicting eukaryotic Pol II promoter sequences. Intronic miRNA clusters were directly reserved for transcription along with their host genes.

##### Expression profile filter

miRNAs in the clusters were also filtered according to expressional correlation. Three expression profiles [50-53] were used in the screening. Only miRNAs with a Pearson Correlation Coefficient higher than 0.3 were retained in the cluster.

#### Quantification of the functional coordination

**Figure 4:**
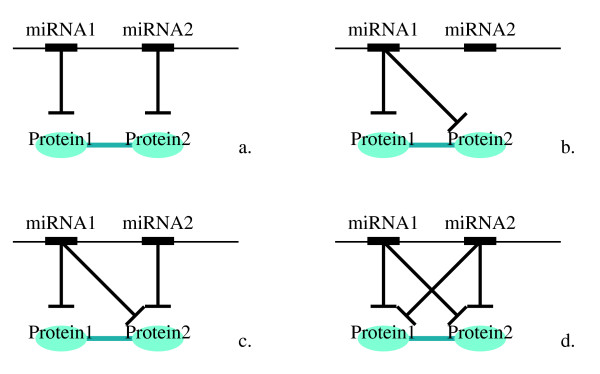
**The four possible ways two proteins can be regulated by a miRNA cluster**.

There are four possible ways two miRNAs in a cluster could target a protein pair (Figure 4). Situation b, concerning the propensity of two interacting proteins to be regulated by the same miRNA, regardless of miRNA clustering, has already be studied by Liang and Li [34]. In our analysis, we therefore excluded this situation. Situation c and d could be caused by the sequence homology of these two miRNAs, whose target sets hence greatly overlap, rather than true coordination. To eliminate the influence of sequence homology, we specifically measured the coordination among different miRNA families, where sequence homology is limited. We constructed a family-represented version of hetero-clusters by mapping miRNAs in a cluster to their families, and having redundancies removed. We separately performed the computation with family-represented hetero-clusters, and employed randomization as control.

#### Randomized Control

In our research, we have performed two types of randomization to construct control set: one is *randomization of miRNA clusters*. To obtain random controls for testing the propensity of interacting proteins to be regulated by sc-miRNAs, we generated a cluster set by randomly shuffling miRNAs among clusters, while keeping the size of each cluster unchanged. The other is *randomization of miRNA-target protein associations*. To obtain random controls for testing the proximity of sc-miRNAs' targets, we generated targets for each miRNA by randomly shuffling the miRNA-target protein associations, while keeping unchanged the number of proteins that a miRNA targets. The empirical P-value for target interactions was calculated against 10,000 independent randomized samples. The negative logarithm of P-value (-*lgP*) was then used to assess the coordination. The greater -*lgP *is, the stronger the coordination is.

#### Others

All the statistical tests and kernel density estimation were done in R. Connectivity and the shortest paths between each protein were calculated using iGraph package .

## Authors' contributions

XY, CL and PY conceived of the study and carried out data analysis. YZ participated and supervised the study. SH participated in the identification of microRNA clusters. QL and SK participated in the study with useful suggestions. XY drafted the manuscript. All authors read and approved the final manuscript.

## Supplementary Material

Additional file 1 **Novel miRNA loci in human, mouse and rat genomes**. This files contains the novel miRNA loci we found in human, mouse and rat genomes. We found them through blasting all pre-miRNA sequences of the three species on their whole genomes. These novel loci were used later to identify miRNA clusters. Click here for file

Additional file 2 **miRNA clusters found in human, mouse and rat genomes**. This files contains two parts. Table 2a contains the miRNA clusters we found in human, mouse and rat genomes and their positions. Table 2b contains the miRNA clusters in which miRNAs have been mapped to their families according to miRBase's assignment. In this table 17 miRNA clusters, in which we have found significant coordination among miRNAs, are marked with red asterisk. Literature supports for the function of 15 of them are also listed. Click here for file

Additional file 3 **miRNA clusters' conservation among human, mouse and rat**. This file contains miRNA clusters conserved or partially conserved among human, mouse and rat. Click here for file

Additional file 4 **Robustness on other data sets**. This file contains the analysis performed on PicTar, TargetScan and PicTar overlapping, as well as miRanda predicted target set. It also contains significant KEGG human pathways that are enriched with target genes of miRNA clusters.
Click here for file
