# Supplementary material for: Clustered microRNAs' coordination in regulating protein-protein interaction network
Source: BMC Syst Biol. 2009 Jun 26;3:65. doi: 10.1186/1752-0509-3-65 (PMC2714305; doi:10.1186/1752-0509-3-65)
Supplement: Additional file 4 — Robustness on other data sets. This file contains the analysis performed on PicTar, TargetScan and PicTar overlapping, as well as miRanda predicted target set. It also contains significant KEGG human pathways that are enriched with target genes of miRNA clusters. [file 1752-0509-3-65-S4.doc]

**Supplementary Table 4: Analysis using PicTar set**

PicTar prediction was downloaded from UCSC genome browser pictar miRNA track.

Target sites density predicted by PicTar is 8.5672$\pm$9.98 in GSN, and 9.37$\pm$8.52 in GSP

**The existence of coordination between sc-miRNAs** GSP-HC is shown in solid line, GSN-HC in dashed line, GSP-RC in solid curve and GSN-RC in dashed curve. The first row is calculated on family-represented hetero-clusters and their 1000 randomizations, and the second row is calculated on original clusters.

**Correlation between coordination and shortest distance**: Spearman’s rank correlation, Rs=-0.132, P< 2.2e-16

**Correlation between coordination and connectivity sum.** 2500 pairs were randomly sampled from GSP and GSN separately. Use Spearman’s rank test for correlation between the sum of the two proteins’ connectivity and the number of clusters that regulate the pair. The last two columns show the results calculated on a random cluster set.

| Spearman | | GSP-HC | GSN-HC | GSP-RC | GSN-RC |
| --- | --- | --- | --- | --- | --- |
| Hetero-Clusters | Rs | 0.144 | 0.025 | 0.06 | 0.0045 |
|  | P | 4.176e-13 | 0.3055 | 0.0026 | 0.8512 |
| All Clusters | Rs | 0.186 | 0.082 | 0.064 | 0.05 |
|  | P | <2.2e-16 | 0.0007 | 0.0012 | 0.0386 |

**Supplementary Table 5: Analysis using TargetScan and PicTar overlapping set**

**The existence of coordination between sc-miRNAs** GSP-HC is shown in solid line, GSN-HC in dashed line, GSP-RC in solid curve and GSN-RC in dashed curve. The first row is calculated on family-represented hetero-clusters and their 1000 randomizations, and the second row is calculated on original clusters.

**Correlation between coordination and shortest distance**: Spearman’s rank correlation, Rs=-0.259, P< 2.2e-16

**Correlation between coordination and connectivity sum.** 2500 pairs were randomly sampled from GSP and GSN separately. Use Spearman’s rank test for correlation between the sum of the two proteins’ connectivity and the number of clusters that regulate the pair. The last two columns show the results calculated on a random cluster set.

| Spearman | | GSP-HC | GSN-HC | GSP-RC | GSN-RC |
| --- | --- | --- | --- | --- | --- |
| Hetero-Clusters | Rs | 0.126 | 0.03 | 0.043 | 0.058 |
|  | P | 2.853e-10 | 0.2186 | 0.0475 | 0.0171 |
| All Clusters | Rs | 0.121 | 0.058 | 0.08 | 0.067 |
|  | P | 1.189e-9 | 0.03 | 0.0007 | 0.013 |

**Supplementary Table 6: Analysis using miRanda set**

miRanda’s prediction in Homo sapiens for 418 miRNAs in microRNA registry release 10.0 were downloaded from microRNA.org Jan 2008 release, with align score bigger than 155.

Target sites density predicted by miRanda is $8.43\pm8.1$ in GSN, and $8.68\pm8.1$ in GSP

**The existence of coordination between sc-miRNAs** GSP-HC is shown in solid line, GSN-HC in dashed line, GSP-RC in solid curve and GSN-RC in dashed curve. The first row is calculated on family-represented hetero-clusters and their 1000 randomizations, and the second row is calculated on original clusters.

**Correlation between coordination and shortest distance**: Spearman’s rank correlation, Rs=-0.155, P<2.2E-16

**Correlation between coordination and connectivity sum.** 2500 pairs were randomly sampled from GSP and GSN separately. Use Spearman’s rank test for correlation between the sum of the two proteins’ connectivity and the number of clusters that regulate the pair. The last two columns show the results calculated on a random cluster set.

| Spearman | | GSP-HC | GSN-HC | GSP-RC | GSN-RC |
| --- | --- | --- | --- | --- | --- |
| Hetero-Clusters | Rs | 0.160 | 0.003 | 0.045 | 0.029 |
|  | P | 9.995e-16 | 0.909 | 0.025 | 0.2405 |
| Ori- Clusters | Rs | 0.100 | 0.039 | 0.069 | 0.042 |
|  | P | 5.943e-7 | 0.1223 | 0.0005 | 0.0916 |

**Supplementary Table 7: Analysis using KEGG Human Pathway Data**

**Significant pathways that are enriched with target genes of miRNA clusters.** Similar to the procedure used in network community analysis, after filtering with the criteria of size bigger than 10, at least 50% of the proteins in the pathway are targeted by a miRNA cluster and at most 40% regulated by any single miRNA of the cluster, we obtained 11 pathway-cluster pairs. We chose the P-value smaller than 0.03 as significant.

| Chr | Cluster | KEGG Human Pathway | Description |
| --- | --- | --- | --- |
| 14(+) | mir-379 | hsa04520 | Adherens junction |
| 14(+) | mir-379 | hsa05210 | Colorectal Cancer |
| X(-) | mir-450 | hsa00471 | D-glutamin and D-glutamin metabolism |
